# Characterization of Fe-Containing and Pb-Containing Nanoparticles Resulting from Corrosion of Plumbing Materials in Tap Water Using a Hyphenated ATM-DMA-spICP-MS System

**DOI:** 10.1021/acs.est.3c07592

**Published:** 2024-01-19

**Authors:** Jing-Wen Wang, Chia-Hung Yu, Wen-Che Hou, Ta-Chih Hsiao, Yi-Pin Lin

**Affiliations:** † Graduate Institute of Environmental Engineering, National Taiwan University, No. 1, Sec. 4, Roosevelt Road, Taipei 10617, Taiwan; ‡ Department of Environmental Engineering, National Cheng Kung University, No. 1 University Road, Tainan City 70101, Taiwan; § NTU Research Center for Future Earth, National Taiwan University, No. 1, Sec. 4, Roosevelt Road, Taipei 10617, Taiwan

**Keywords:** metallic nanoparticles, spICP-MS, lead, iron, tap water

## Abstract

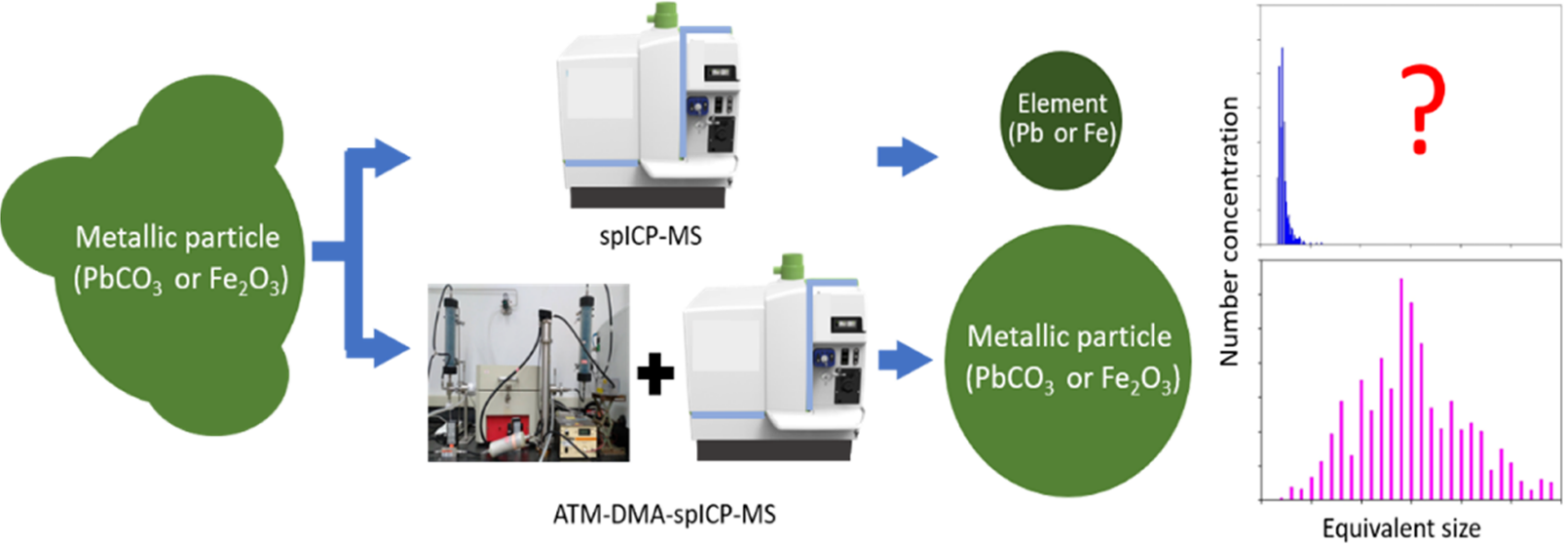

Single-particle inductively coupled plasma mass spectrometry (spICP-MS) has been used to characterize metallic nanoparticles (NPs) assuming that all NPs are spherical and composed of pure element. However, environmental NPs generally do not meet these criteria, suggesting that spICP-MS may underestimate their true sizes. This study employed a system hyphenating the atomizer (ATM), differential mobility analyzer (DMA), and spICP-MS to characterize metallic NPs in tap water. Its performance was validated by using reference Au nanoparticles (AuNPs) and Ag-shelled AuNPs. The hyphenated system can determine the actual size and metal composition of both NPs with additional heating after ATM, while stand-alone spICP-MS misidentified the Ag-shelled AuNPs as smaller individual AgNPs and AuNPs. Dissolved metal ions could introduce artifact NPs after heating but could be eliminated by centrifugation. The hyphenated system was applied to characterize Fe-containing and Pb-containing NPs resulting from the corrosion of plumbing materials in tap water. The mode sizes of Fe-containing and Pb-containing NPs were determined to be 110 and 100 nm and the particle number concentrations were determined to be 4.99 × 10^7^ and 1.40 × 10^6^ #/mL, respectively. Cautions should be paid to potential changes in particle size induced by heating for metallic NPs with a low melting point or a high organic content.

## Abstract 2

The sizes and concentrations of Fe-containing and Pb-containing nanoparticles in tap water resulting from the corrosion of plumbing materials can be determined using the hyphenated ATM-DMA-spICP-MS system.

## 1 Introduction

Metallic nanoparticles (NPs) have been widely used in industrial processes and consumer products and can be released into the environment.^1,2^ They can absorb, coprecipitate, or trap other contaminants and change their fate and transport in the environment.^3^ The unique physical and chemical properties of metallic NPs resulting from their nanoscale size and large surface area to volume ratio could make them more toxic.^3,4^ It has been reported that metallic NPs may damage the cell membrane, disrupt DNA replication and ATP production, alternate microbial gene expressions, and cause embryonic fatality in fish.^5−7^ Different metallic NPs have been found in wastewater treatment plants, rivers, and lakes.^8−11^ Metallic NPs can also be found in tap water due to the corrosion of plumbing materials^12^ and can enter the human body via drinking water consumption.^13−17^ Specifically, the presence of lead-containing NPs in tap water due to the corrosion of aged lead pipes and lead-containing plumbing materials in the distribution system has aroused public concern.^18,19^ Analytical methods that can comprehensively characterize metallic NPs in tap water are crucial in monitoring their presence and developing treatment strategies to control their release.

Different analytical methods, including scanning electron microscopy (SEM), transmission electron microscopy (TEM), dynamic light scattering (DLS), and nanoparticle tracking analysis (NTA), have been employed for NPs characterization.^20^ Microscopic methods can provide images for direct observation of a limited number of samples, and DLS and NTA can reveal the particle size distribution (PSD) in an arbitrary scale. In the past decade, an advanced mode of inductively coupled plasma mass spectrometry (ICP-MS), named single-particle ICP-MS (spICP-MS), has been extensively applied to simultaneously determine the particle mass, size distribution, and number concentration of metallic NPs.^21^ Nonetheless, the particle size is converted from the particle mass and the density of the target element, assuming that all metallic NPs are spherical and composed of only the target element, which is not true for most metallic NPs of interest. Therefore, inherent errors exist for complex environmental samples. For example, Venkatesan et al.^17^ used spICP-MS to investigate the presence of NPs containing Pb, Fe, Sn, and Cu in tap water samples resulting from the corrosion of plumbing materials in the distribution system. However, from the TEM and energy dispersive X-ray (EDX) elemental analysis, it could be found that the particles were nonspherical and were not pure metallic NPs, suggesting that the spICP-MS method may underestimate their true sizes. Moens et al.^22^ also cautioned the results when applying spICP-MS to determine the size of colloids or coated metallic NPs.

In order to better characterize complex NPs, methods integrating particle size separation and spectroscopic detection have been proposed.^23^ Among them, the system hyphenating differential mobility analyzer (DMA) with spICP-MS demonstrated a unique capability.^24,25^ Instead of directly analyzing the liquid sample, the system first transformed the sample into airborne using aerosol generators, such as electrospray (ES)^24,26,27^ or atomizer (ATM).^28−30^ With the assistance of DMA, the aerosol particles within a narrow geometric size range can then be selected before determining their chemical compositions by the following spICP-MS. Although ES can generate fine monodispersed droplets, the high charging status could result in excess particle loss and the high-voltage electrical field restricts the use of argon as the carrying gas. On the other hand, ATM does not exhibit the drawbacks mentioned above, while the potential aggregation of NPs in either liquid or air phases could be critical for the ionization process by the inductively coupled plasma.^29^ Another potential drawback of the ATM is the introduction of more dissolved impurities due to the larger droplets it generates.

In this study, a hyphenated ATM-DMA-spICP-MS system was established to characterize Fe-containing and Pb-containing NPs in tap water. The system was first validated using reference AuNPs and Ag-shelled AuNPs and then applied to characterize Fe-containing and Pb-containing NPs present in tap water. Finally, the results were compared head-to-head to those obtained from stand-alone spICP-MS to demonstrate the superior capability of the hyphenated ATM-DMA-spICP-MS system in characterizing metallic NPs in environmentally relevant conditions.

## 2 Materials and Methods

### 2.1 Configuration of the Hyphenated ATM-DMA-spICP-MS System

**Figure 1:**
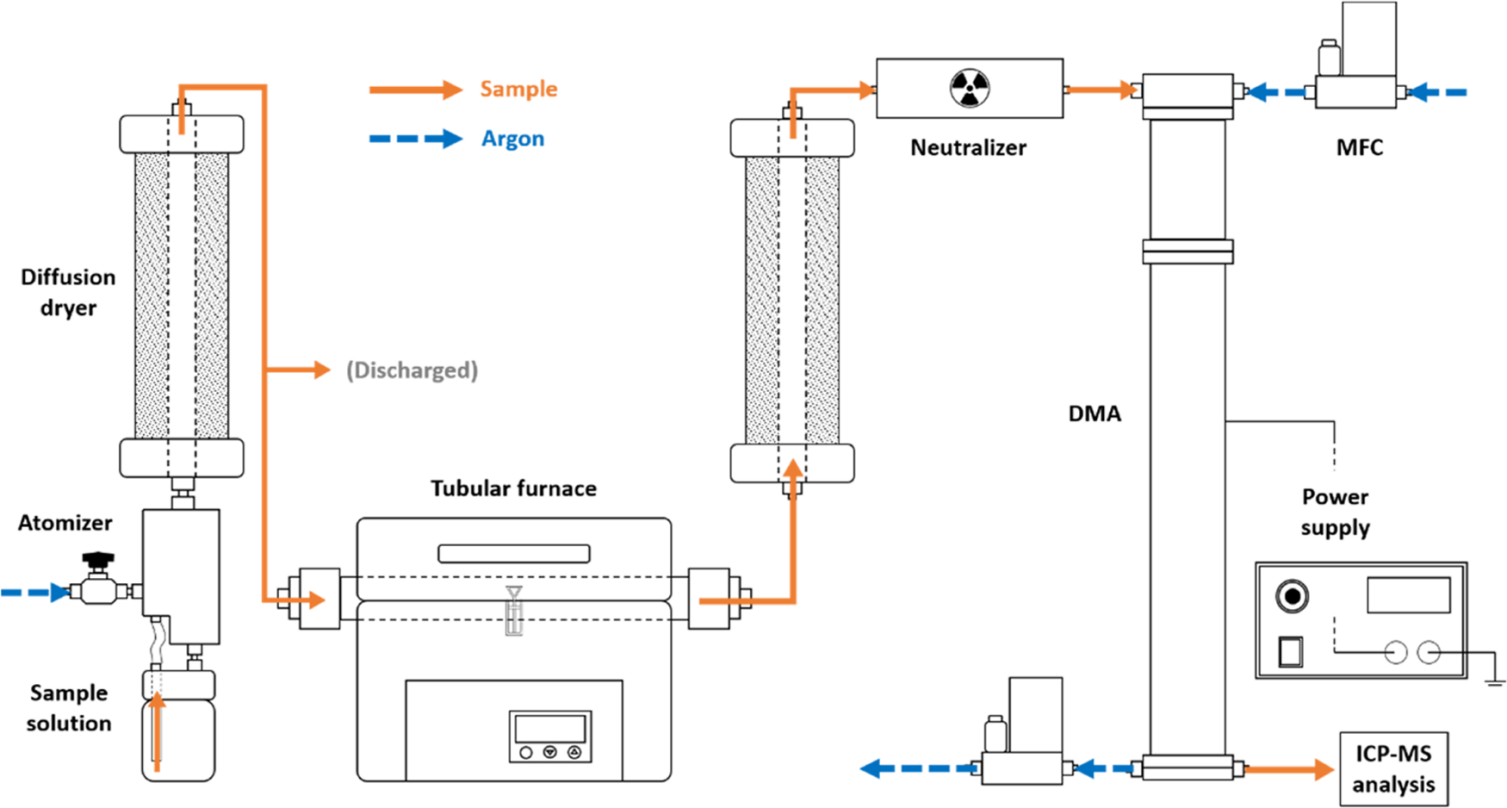
Schematic of the hyphenated ATM-DMA-spICP-MS system.

The ATM-DMA-spICP-MS hyphenated system comprises three major parts: the aerosol generator, DMA, and spICP-MS. The schematic of the system is shown in Figure 1. In this study, microdroplets, containing suspended NPs, were generated by an atomizer-type aerosol generator (model 3076, TSI) before passing through the diffusion dryer to remove water content. A tubular furnace (T11–301, SJ, Taiwan) was installed to solve the “tailing” phenomena observed in the data obtained from the hyphenated system, which is discussed later. Following the heating by the furnace, the aerosol particles pass through an ^85^Kr neutralizer to reach the Boltzmann distribution (predominantly +1, 0, and −1 charges) before being classified by the DMA, in which the midpoint electrical mobility was selected by adjusting the voltage provided by the power supply (230–10R, Spellman Bertan, 10 kV maximum). The upper operation limit was set at 3500 V to prevent arcing of argon. Two types of DMA, long-DMA (model 3081, TSI; effective L: 44.4 cm) and nano-DMA (model 3085, TSI; effective L: 5.0 cm), were used for different operating ranges of particle size classification. For both DMAs, the sheath flow and the aerosol flow were controlled at 3 and 0.3 L/min, respectively, by the mass flow controllers (MFC) (5800E, Brooks). The classified aerosol particles were then sent directly into the spray chamber of ICP-MS (NexION2000, PerkinElmer) without secondary nebulization. Since the entering aerosol sample flow was only 0.3 L/min, a 0.8 L/min auxiliary argon flow was provided to ensure analytical stability.

### 2.2 Operation of the Stand-Alone spICP-MS and Hyphenated ATM-DMA-spICP-MS System

For stand-alone spICP-MS, metallic nanoparticles enter the plasma and become ionized. These ions are detected as individual pulses above the background signal, and each pulse represents a single nanoparticle. The particle number concentration (*C_n_*) is obtained from the pulse counting (*N*), transport efficiency (η), scanning time of spICP-MS (*t*), and liquid sample flow rate entering the spICP-MS (*Q*) using the following equation:
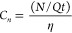
1

For stand-alone spICP-MS, the transport efficiency is the ratio of the number of particles detected to that entering the spICP-MS. It was determined each time before sample analysis. Table S1 shows the range of transport efficiency for stand-alone spICP-MS in selected experiments when 50 nm reference AuNPs solution with a number concentration of 4 × 10^4^ #/mL was employed. The dwell time of spICP-MS was 100 μs. The transport efficiency was determined to range from 4.14 to 9.34%, with an average of 6.27% (σ = 1.23%, *n* = 23). The calibration curve for particle size detection was constructed using soluble gold (High-purity Standards) or multielement standard (AccuStandard) with different mass concentrations. The results were presented using the number-based particle size distribution (PSD). The raw output data for stand-alone spICP-MS were converted to mass equivalent diameter (*D*_m_), assuming that the particle was spherical and composed of the target metal only using the software provided by the instrument manufacturer.

For the ATM-DMA-spICP-MS hyphenated system, the midpoint electrical mobility selected by the DMA was increased stepwisely and maintained at each voltage for a sufficient period for the following spICP-MS analysis (20 s for the sample transmission from DMA to spICP-MS interface and 30 s for elemental analysis in spICP-MS). The results were obtained by “scanning” the midpoint electrical mobility throughout the whole range of size distribution. The particle number concentration was determined using eq 2.
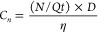
2where *Q* represents the gas sample flow rate and *D* is a parameter considering the sample conversion from liquid phase to gas phase in ATM (1 mL/min liquid sample mixed with 1.7 L/min Ar gas), the gas sample bypass when exiting DMA (0.3 L/min out of 1.7 L/min entering the spICP-MS) and the dilution in spICP-MS (0.3 L/min gas sample mixed with 0.9 L/min auxiliary gas). *D* was determined to be 38.5 L of gas/mL liquid.

The calculation is similar to that used for stand-alone spICP-MS except that the transport efficiency in the hyphenated system is the ratio of the number of particles detected to those entering the hyphenated system.

Table S2 shows the range of transport efficiency for the hyphenated system in selected experiments when 50 nm reference AuNPs solutions with a number concentration ranging from 10^7^ to 10^8^ #/mL were used. The transport efficiency was determined to range from 1.00 to 1.86%, with an average of 1.35% (σ = 0.26%, *n* = 20). The particle size was predetermined by the DMA-selected mobility diameter (*D*_e_). The results were compared with the *D*_m_ determined using stand-alone spICP-MS.

### 2.3 Materials and Chemicals

The AuNPs used in this study were purchased from PerkinElmer (N8142300, nominal diameter = 30 nm; N8142302, nominal diameter = 50 nm) and NanoComposix (SCM0090, nominal diameter = 80 nm). The Ag-shelled AuNPs with a nominal size of 60 nm were obtained from NanoComposix (BMCH60, Au core diameter = 30 nm and Ag shell thickness = 15 nm). These NPs were characterized using TEM (Hitachi-7650, Japan), and their diameters were comparable to the nominal diameters provided by the manufacturers (Figure S1). These NPs were coated with citrate to prevent agglomeration. Deionized water produced by the PURelAB classic system (ELGA, U.K.) was used for sample preparation and dilution.

The tap water sample was collected from a building on the National Taiwan University campus using 1 L HDPE bottles on February 03, 2023. For total metal concentration analysis, a 10 mL subsample was withdrawn from the well-mixed tap water sample and digested for 2 h at 85 °C with 5% v/v nitric acid, followed by the ICP-MS analysis. For soluble metal concentration analysis, a separate subsample was filtered using a 0.22 μm pore size poly(vinylidene fluoride) (PVDF) filter membrane before metal analysis. It should be noted that the soluble metal was operationally defined. The sample was then spiked with the 50 nm reference AuNPs (particle number concentration = 4 × 10^7^ #/mL) before centrifugation and NPs analysis using the stand-alone spICP-MS and the ATM-DMA-spICP-MS hyphenated system. All samples and AuNPs reference solutions were sonicated for 2 min, followed by vortex mixing before withdrawal for sample preparation or spiking.

## 3 Results and Discussion

### 3.1 Performance Evaluation of the ATM-DMA-spICP-MS Hyphenated System

**Figure 2:**
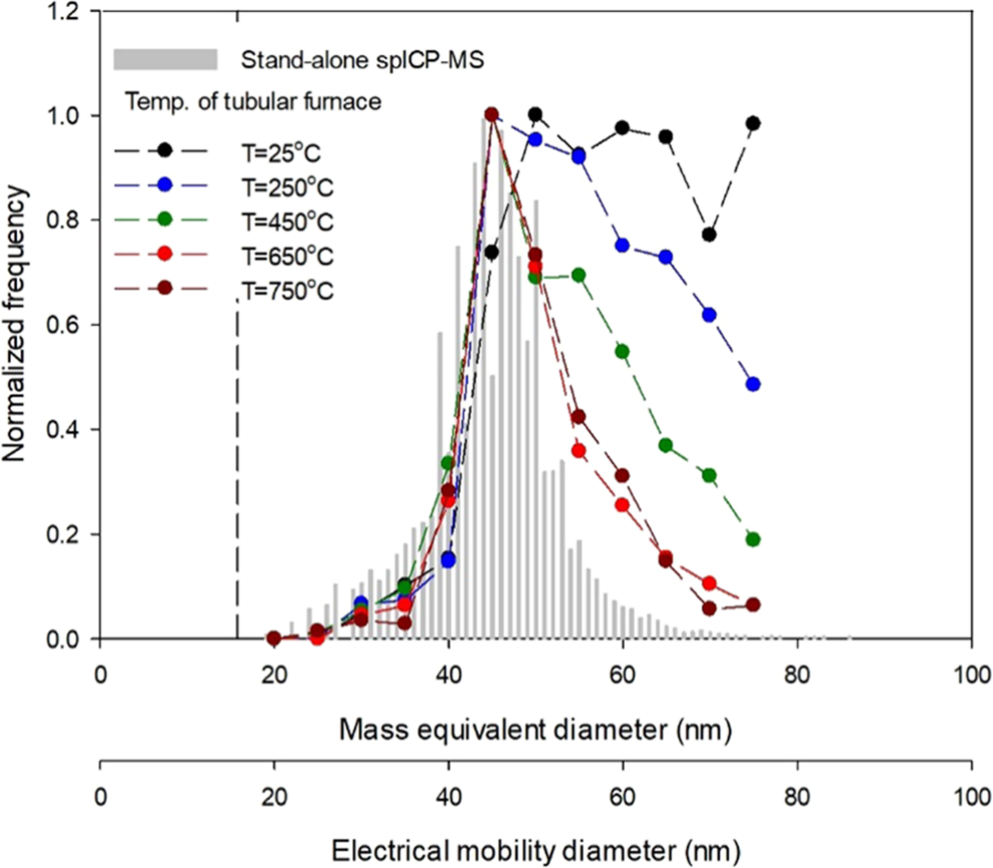
PSD of 50 nm reference AuNPs obtained from the ATM-DMA-spICP-MS hyphenated system at different temperatures of the tubular furnace. The SDL (16 nm) is labeled as the dashed line.

Reference AuNPs with known nominal sizes were employed to evaluate the sizing ability of the ATM-DMA-spICP-MS hyphenated system. Since the reference AuNPs were pure spherical Au particles, it was expected that the PSDs obtained by the stand-alone spICP-MS and the hyphenated system should be identical. Thus, the former was used as the reference to evaluate the performance of the hyphenated system, as shown in Figure 2. First, the evaluation was conducted using 50 nm reference AuNPs at ambient temperature (25 °C). The result, however, indicated that the PSD obtained from the ATM-DMA-spICP-MS hyphenated system (black line in Figure 2) was inconsistent with that obtained from the stand-alone spICP-MS (histogram in Figure 2). The number of particles counted by the ATM-DMA-spICP-MS hyphenated system was higher than expected when the DMA selecting size exceeded the nominal size (50 nm) of the reference AuNPs, resulting in a “tail” behind the theoretical peak of the PSD. A similar phenomenon has been observed by Hsieh et al.,^25^ and this is likely due to particle aggregation caused by water bridges. Thus, a tubular furnace was installed to heat the aerosols before DMA to remove the water. The PSDs with different operating temperatures are also presented in Figure 2. With increasing temperature, the PSDs became narrower and eventually overlapped with the reference. The optimal operating temperature was determined to be 650 °C. The size detection limit (SDL) was determined using the method proposed by Lee et al.^31^ The SDL of the stand-alone spICP-MS and the ATM-DMA-spICP-MS hyphenated system were both determined to be 16 nm at 25 and 650 °C, shown as the dashed lines in the figure.

It should be noted that, however, using heating to reduce nanoparticle aggregation may have two potential impacts: (1) Formation of artifact nanoparticles caused by precipitation of dissolved metal ions if they are present in the sample and (2) change of particle size if the metallic NPs melt or the metallic NPs are linked by organic polymer such as dissolved organic matter or contain a high organic fraction. The first limitation can be eliminated if the dissolved metal ions can be removed prior to the ATM-DMA-spICP-MS measurement. This will be demonstrated using centrifugation, as shown later. As for the potential change of particle size for metallic NPs with a low melting point, the residence time of the sample in the furnace was around 40 s (volume of the effective tubular furnace = 196 cm^3^, gas flow rate = 5 cm^3^/s). After leaving the furnace, the sample temperature quickly returned to room temperature due to the use of a water cooling system. We expect that the water surrounding the metallic NPs would evaporate quickly inside the furnace. However, the melting of metallic NPs would not be significant due to the short residence time. Even the metallic NPs do melt to some extent inside the furnace, they will quickly solidify upon exiting the furnace due to water cooling, and their sizes are expected to be similar without significant change. As for metallic NPs linked by organic polymer or containing a high organic fraction, the particle size could indeed be altered after the organic polymer or organic fraction is destroyed by the high temperature, which presents a limitation of the proposed method. Cautions should be given to such samples.

**Figure 3:**
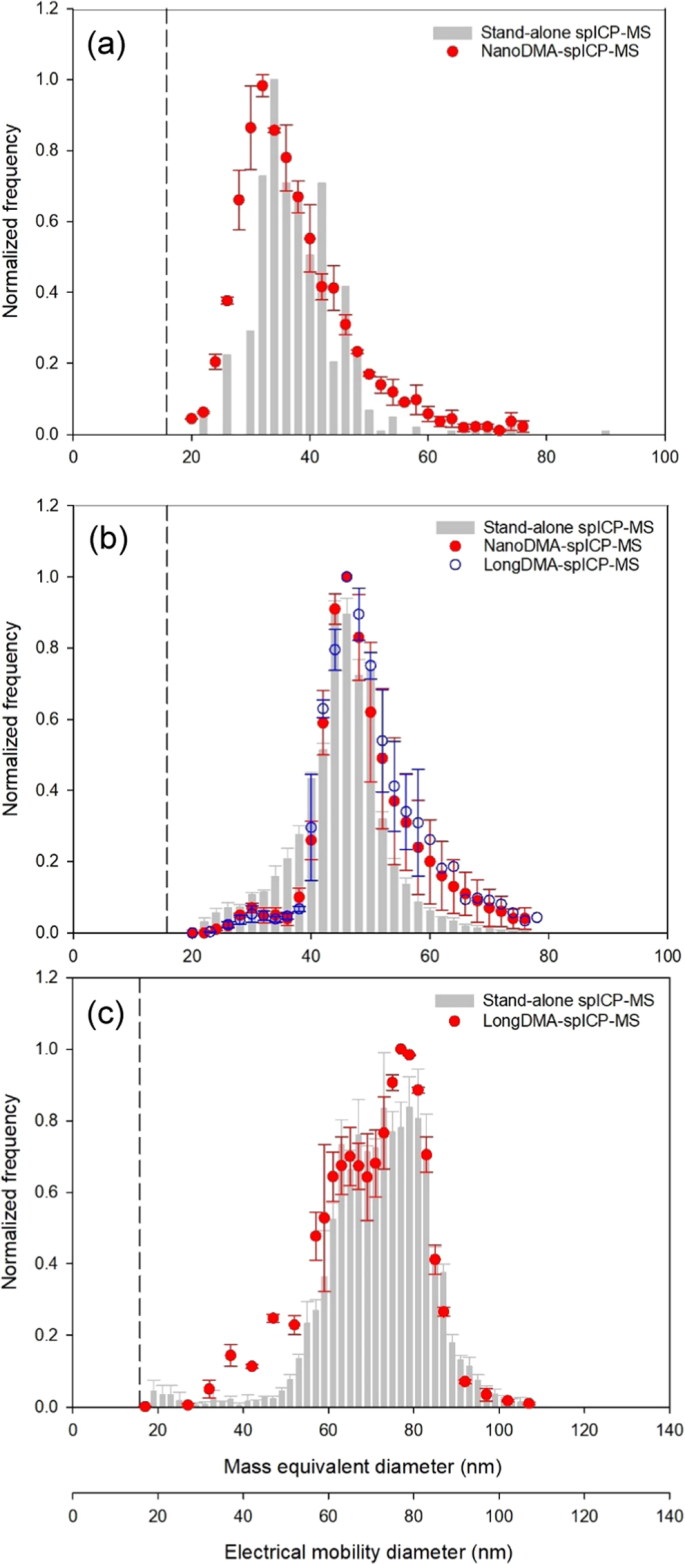
PSDs from the stand-alone spICP-MS and the hyphenated system for reference AuNPs with a nominal size of (a) 30 nm, (b) 50 nm, and (c) 80 nm.

The performance of the ATM-DMA-spICP-MS hyphenated system was further evaluated by using (a) 30 and 50 nm reference AuNPs when the system was equipped with the nano-DMA and (b) 50 and 80 nm reference AuNPs when the system was equipped with the long-DMA. The obtained PSDs are shown in Figure 3, and those obtained by the stand-alone spICP-MS are also shown. The determined mode size and full width at half-maximum (FWHM) for each reference AuNP are summarized in Table 1. For all reference AuNPs, the mode size and associated FWHM obtained from the ATM-DMA-spICP-MS hyphenated system were comparable to those obtained from the stand-alone spICP-MS (difference <2 nm), except that a relatively large deviation (6 nm) was found for the FWHM of 30 nm reference AuNP. The deviation could be related to the DMA transfer function, which became wider as the midpoint mobility increased (i.e., particle size decreased) due to particle diffusion.^32^ Also, it is noted that for measurements of 50 nm reference AuNP using the hyphenated system, a minor peak around 30 nm was observed, which could be attributed to the double charges possessed by a small fraction of 50 nm AuNPs. Nevertheless, the differences in sizing performance between the stand-alone spICP-MS and the hyphenated system were within 6%, suggesting that the ATM-DMA-spICP-MS hyphenated system equipped with either nano-DMA or long-DMA can provide acceptable results for particle size characterization.

**Table 1:** Mode Size and FWHM of the Reference AuNPs Determined Using Stand-Alone spICP-MS and ATM-DMA-spICP-MS Hyphenated System

reference AuNPs	method	mode size (nm)	FWHM (nm)
30 nm	stand-alone spICP-MS	34	9
	ATM-DMA-spICP-MS (nano-DMA)	32	15
50 nm	stand-alone spICP-MS	44	10
	ATM-DMA-spICP-MS (long-DMA)	46	10
	ATM-DMA-spICP-MS (nano-DMA)	45	10
80 nm	stand-alone spICP-MS	79	24
	ATM-DMA-spICP-MS (long-DMA)	77	26

### 3.2 Upper Limit of Particle Number Concentration

The upper limits of particle number concentration that can be detected by the stand-alone spICP-MS and the hyphenated systems are different. When a sample contains a particle number concentration exceeding the upper limit that can be detected by the method, the obtained mode size would be bigger than the actual mode size because the particles are not well-separated, and more than one particle would be present within the dwell time and counted as a bigger particle. Dilution is required for such samples.

Table S3 shows the results for the determination of the upper limit particle number concentration for the stand-alone spICP-MS using 50 nm reference AuNPs. The results indicated that as the particle number concentration increased, the obtained mode size shifted toward a larger size. Specifically, when the particle number concentration was <2 × 10^5^ #/mL, the determined mode size ranged from 52 to 54 nm, which was close to the nominal size of 50 nm, while the mode size increased to 58–66 nm when the particle number concentration was >2 × 10^5^ #/mL, suggesting that multiple particles were being analyzed within the dwell time. As for the obtained particle number concentration, the recoveries were within 75–125% when the introduced particle number concentration was ≤2 × 10^5^ #/mL. Therefore, the upper limit of particle number concentration for the stand-alone spICP-MS was determined to be 2 × 10^5^ #/mL if the acceptable criterion of mode size deviation was set at ±10% and recovery was set at 75–125%.

Table S4 shows the results for the determination of the upper limit particle number concentration for the hyphenated system using 50 nm reference AuNPs with the particle number concentration ranging from 4 × 10^6^ to 4 × 10^8^ #/mL. The results indicated that the mode size of NPs shifted toward a larger size of 66 nm and the recovery of particle number concentration reached 130% due to a lower transport efficiency (0.83%) when the introduced particle number concentration was 4 × 10^8^ #/mL, which did not meet the above-mentioned criteria of mode size deviation and recovery. Therefore, the upper limit of the hyphenated system was determined to be 2 × 10^8^ #/mL.

### 3.3 Characterization of Shelled Nanoparticle

**Figure 4:**
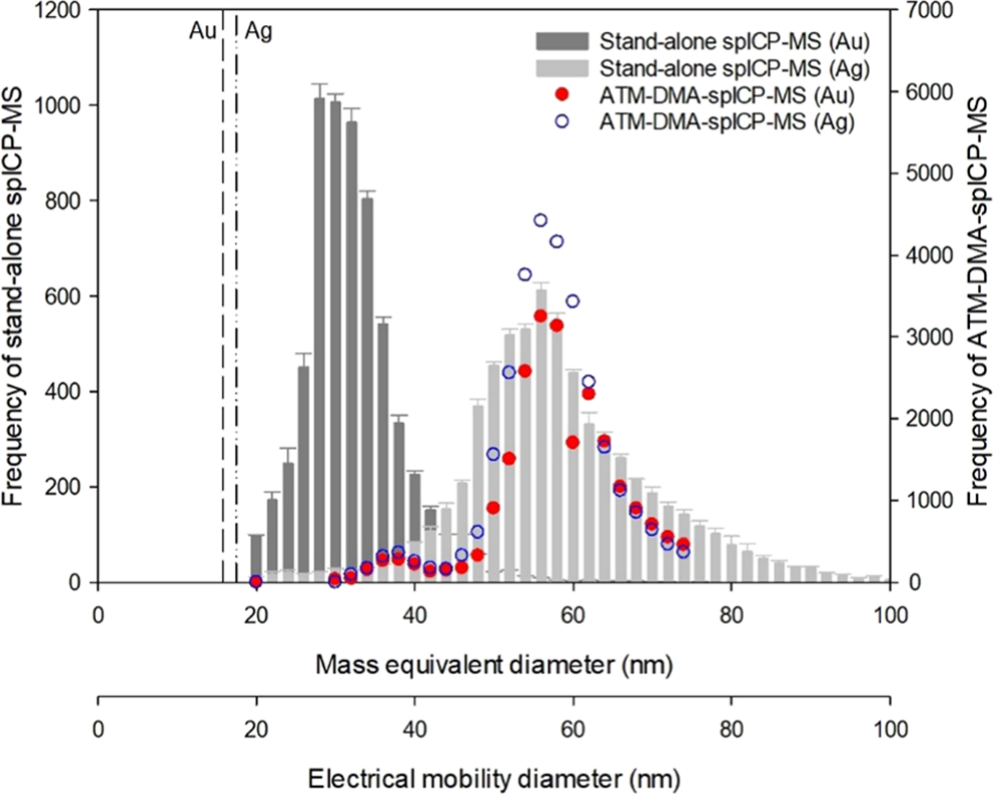
PSDs of reference Ag-shelled AuNPs determined using the stand-alone spICP-MS and the hyphenated system.

As mentioned initially, metallic NPs in the environment cannot be as perfect as a spherical particle made of a single element. Therefore, the reference Ag-shelled AuNPs were used to test the ability of the hyphenated system to simultaneously determine the physical size and the mass equivalent size of metallic NPs composed of multiple elements. The Ag-shelled AuNPs with a nominal size of 60 nm were analyzed using the stand-alone spICP-MS and the hyphenated system equipped with the nano-DMA. Given that the particle size is determined based on the mass and density of the known element in the stand-alone spICP-MS measurements, two separated Ag and AuNPs, instead of one bigger particle mixed of Ag and Au, are expected to be found for the Ag shell AuNPs when stand-alone spICP-MS is employed. As the Ag shell thickness was 15 nm and the Au core diameter was 30 nm, the diameter of a mass equivalent Ag sphere would be 57 nm, and that for the Au core would be kept unchanged. As shown in Figure 4, two distinct Ag and Au peaks with the predicted mode *D*_m_ were determined in the stand-alone spICP-MS measurements. This result revealed that the size information provided by the stand-alone spICP-MS is questionable if the metallic NPs comprise multiple elements. In other words, the stand-alone spICP-MS data can be interpreted only under the externally mixed assumption, while the internally mixed condition is more general for environmental samples.

On the other hand, the ATM-DMA-spICP-MS hyphenated system selects the particle size before analyzing the chemical composition. Similar PSDs with a mode *D*_e_ of 56 nm, which was close to the nominal size (60 nm) of the reference Ag-shelled AuNPs, were obtained when characterizing both Au and Ag (Figure 4). The results suggested that the sample contained 56 nm particles composed of Au and Ag. According to the frequency obtained by the hyphenated system, the volumetric fractions of Au and Ag in the monodispersed particles were estimated to be about 6.9 ± 0.4 and 93.1 ± 0.4%, respectively. Based on the nominal size provided by the supplier of the Ag-shelled AuNPs, i.e., Au core diameter = 30 nm and Ag shell thickness = 15 nm, the theoretical volume fractions of Au and Ag would be 12.5 and 87.5%, respectively. Although the measured values somehow deviated from the theoretical values, considering the range of particle size observed under TEM (58.9 ± 5.3 nm, Figure S1(d)), these differences were believed to be within an acceptable range. Based on these observations, it is evident that the ATM-DMA-spICP-MS hyphenated system is capable of characterizing the physical size as well as providing the size-resolved composition of metallic NPs.

### 3.4 Interference Caused by Dissolved Ions and Its Elimination

When a water sample contains dissolved ions, artifact nanoparticles may form in the furnace and interfere with nanoparticle characterization by the hyphenated system. Therefore, we evaluated the influence of soluble Au ions on the characterization of 50 nm AuNPs and the effectiveness of using centrifugation to eliminate the interference if it exists.

**Figure 5:**
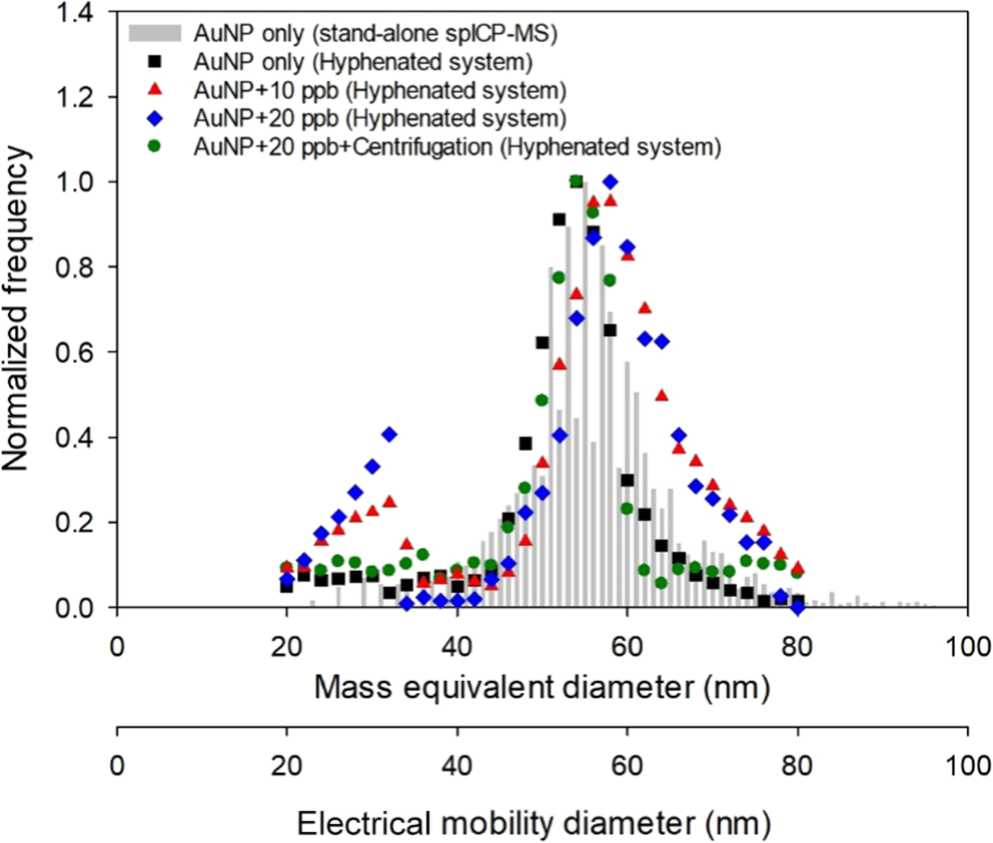
Influences of dissolved Au ions and centrifugation on the PSD of 50 nm reference AuNPs obtained using the stand-alone spICP-MS and the ATM-DMA-spICP-MS hyphenated system.

Figure 5 shows the influences of 10 and 20 ppb spiked Au ions on the PSDs of 50 nm AuNPs determined using the ATM-DMA-spICP-MS hyphenated system. The particle number concentration of AuNPs employed was 4.0 × 10^7^ #/mL. The PSDs for the sample without dissolved Au ions determined by the stand-alone spICP-MS (after 100 times dilution) and the hyphenated system are also shown for comparison purposes. It was found that the FWHM of PSD increased when dissolved Au ions were present, and an unexpected peak at 30 nm appeared for both Au ion concentrations. These results indicated that new artifact nanoparticles could form in the presence of dissolved ions and interfere with the ATM-DMA-spICP-MS measurements. For a separate sample spiked with 20 ppb Au ions, prior centrifugation at 8000 rpm for 15 min followed by replacement of the supernatant with deionized water and sonication was conducted before the hyphenated system analysis. The PSD of the sample is also shown in Figure 5. After centrifugation, the unexpected peak was effectively removed, and the FWHM was similar to the results without dissolved Au ions, demonstrating that centrifugation is an effective pretreatment to eliminate the interference caused by dissolved ions. It should be noted that, however, although centrifugation can effectively eliminate the interferences caused by dissolved ions, some NPs may be lost during this procedure indicated by the lower frequency before normalization (Figure S2). This loss should be taken into consideration when calculating the particle number concentrations in the sample. For example, in Figure S2, the particle number concentration determined for AuNP + Centrifugation (Hyphenated) was 2.2 × 10^7^ #/mL based on the transport efficiency (1.39%) determined using the AuNP only (Hyphenated) result. Therefore, a centrifugation factor of 1.82 (=4.0 × 10^7^/2.2 × 10^7^) should be multiplied by the particle number concentration obtained after centrifugation to determine the actual particle number concentration in the sample.

### 3.5 Application for Fe-Containing and Pb-Containing NPs Analysis in Tap Water Sample

Iron and lead could be present in tap water in dissolved or particulate forms.^12,33−37^ Depending on water chemistry, the corrosion of plumbing materials may result in the formation of corrosion products with different surface morphologies and elemental compositions.^38−40^ Detachments of these corrosion products would contribute to the Fe-containing or Pb-containing NPs in drinking water.^12,41,42^ A 1 L tap water sample was collected on the National Taiwan University campus for the characterization of Fe-containing and Pb-containing NPs. Total iron and total lead concentrations were determined to be 19.9 and 42.3 μg/L and the soluble iron and soluble lead concentrations were determined to be 13.9 and 20.1 μg/L, respectively.

**Figure 6:**
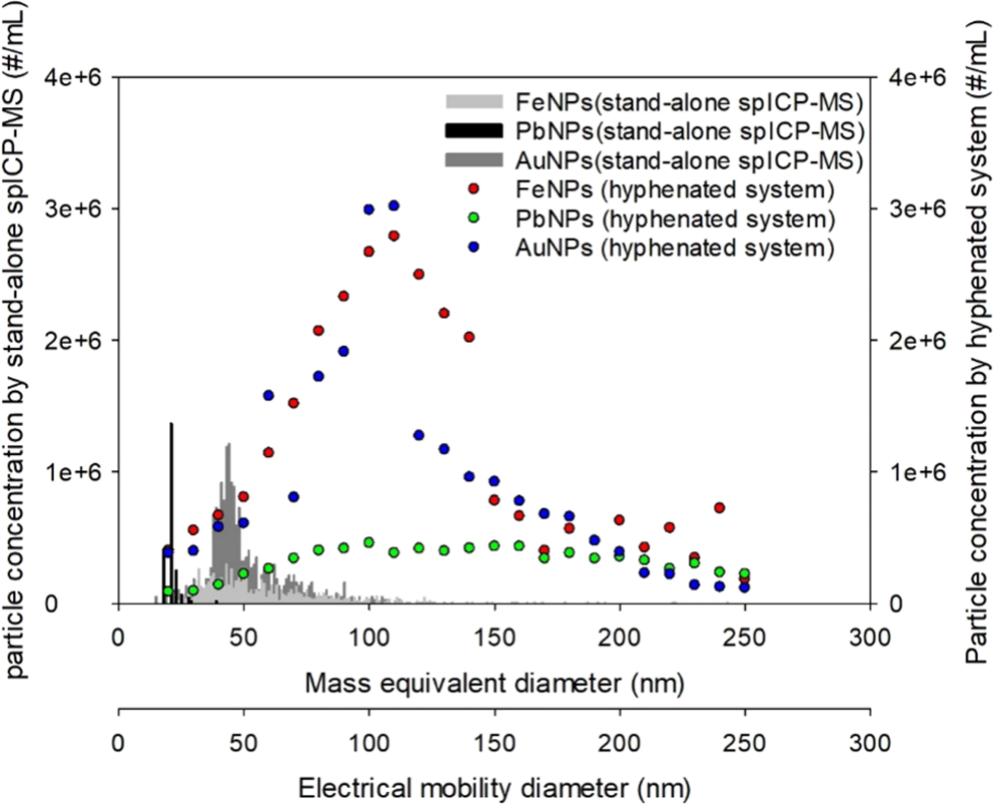
PSDs of AuNPs, Fe-containing NPs, and Pb-containing NPs detected by the stand-alone spICP-MS and the ATM-DMA-spICP-MS hyphenated system.

**Figure 7:**
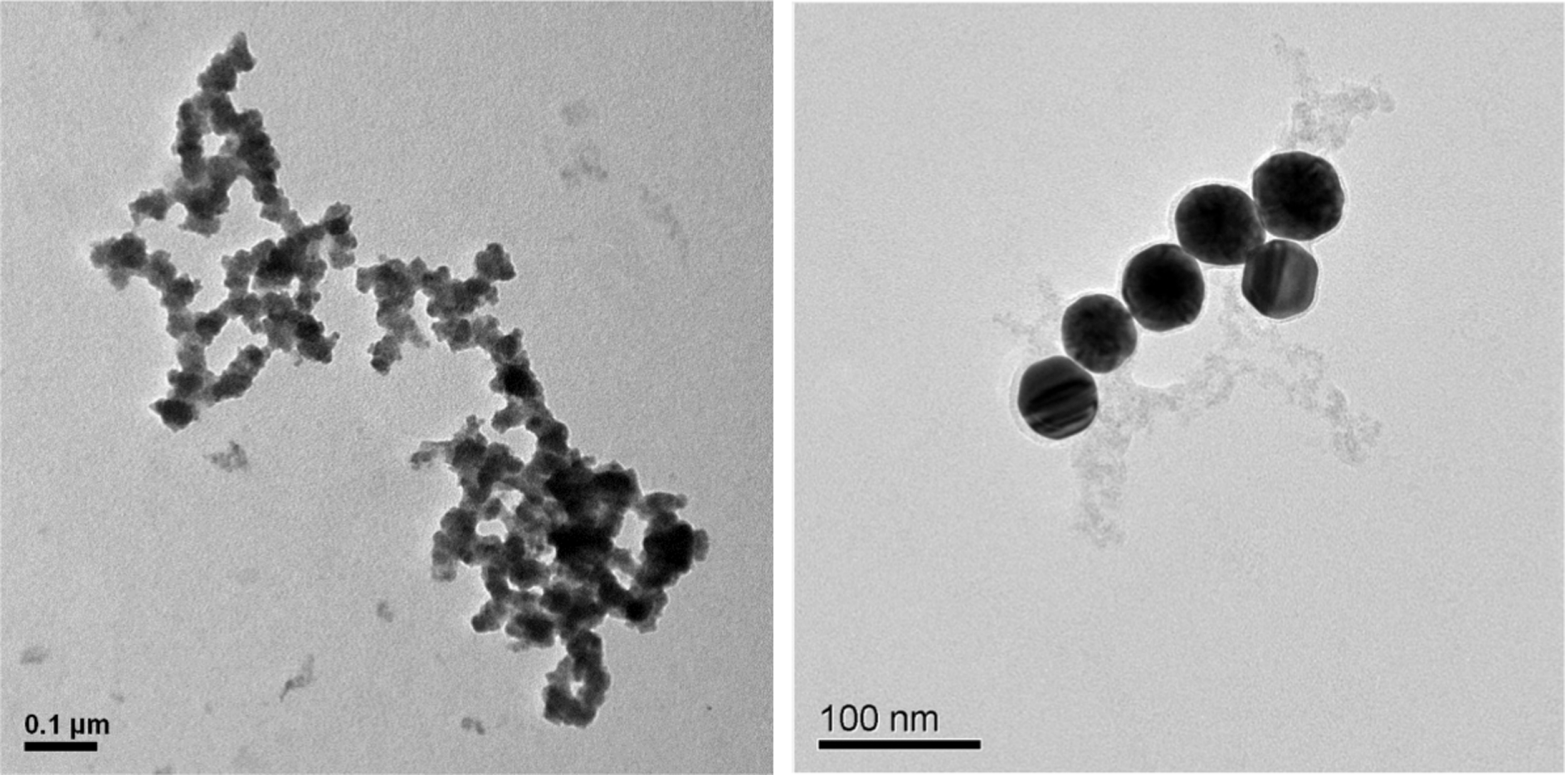
TEM images of NPs collected from drinking water spiked with 50 nm reference AuNPs.

Fe-containing NPs and Pb-containing NPs were quantified using both stand-alone spICP-MS and ATM-DMA-spICP-MS hyphenated systems after the sample was spiked with 4 × 10^7^ #/mL 50 nm reference AuNPs, followed by centrifugation to remove soluble iron and lead ions. For the stand-alone spICP-MS analysis, the sample was diluted 100 times after centrifugation to avoid potential exceedance of the upper limit of particle number concentration (2 × 10^5^ #/mL) as separate experiments using subsamples showed that below this dilution, a larger mode size and a lower particle number concentration were found (Table S5). These results indicated that the particle number concentration was too high below this dilution for the stand-alone spICP-MS measurements. The transport efficiency of stand-alone spICP-MS determined using 4 × 10^4^ #/mL 50 nm AuNPs prior to the sample analysis was 4.17%. For the hyphenated system, the sample was not diluted as the particle number concentrations for Fe-containing NPs and Pb-containing NPs were expected to be in the range of 1 × 10^6^–10^7^ #/mL (Table S5), which did not exceed the maximum number concentration for the hyphenated system. The transport efficiency of the hyphenated system determined using 4 × 10^7^ #/mL 50 nm reference AuNPs was 1.35%. The two transport efficiencies and a centrifugation factor of 1.82 were used to calculate the particle number concentrations with the assumption that the transport efficiency determined using reference AuNPs also applies to Fe-containing NPs and Pb-containing NPs. The PSDs obtained from the two systems are shown in Figure 6. It should be noted that the PSD and mode size of AuNPs obtained by using the ATM-DMA-spICP-MS hyphenated system were similar to those of Fe-containing NPs, suggesting that AuNPs were attached to Fe-containing NPs. The TEM image of the sample revealed the presence of aggregated NPs and the adherence of spiked 50 nm reference AuNPs to these aggregated NPs (Figure 7). The obtained mode sizes and particle number concentrations for AuNPs, Fe-containing NPs, and Pb-containing NPs are summarized in Table 2.

**Table 2:** Mode Size and Particle Number Concentration of Fe-Containing and Pb-Containing NPs Determined Using the Stand-Alone spICP-MS and the ATM-DMA-spICP-MS Hyphenated System

		mode size (nm)	particle number concentration (#/mL)
AuNPs	stand-alone spICP-MS	44	3.30 × 10^7^
	ATM-DMA-spICP-MS	110	4.03 × 10^7^
Fe-containing NPs	stand-alone spICP-MS	43	1.44 × 10^7^
	ATM-DMA-spICP-MS	110	4.99 × 10^7^
Pb-containing NPs	stand-alone spICP-MS	21	3.99 × 10^6^
	ATM-DMA-spICP-MS	100	1.40 × 10^7^

For Fe-containing NPs, the mode sizes determined by the stand-alone spICP-MS and the hyphenated system were 43 (*D*_m_) and 110 nm (*D*_e_), respectively (Table 2). It should be noted that the mode *D_m_* determined by the stand-alone spICP-MS only represents the mass size of the selected metallic element and ignores the contribution of others if multiple elements are present in the particle, while the mode *D*_e_ determined by the ATM-DMA-spICP-MS hyphenated system represents the mobility size of the whole particle composed of more than one element as we demonstrated for the Ag-shelled AuNPs. Therefore, the sizes of Fe-containing NPs determined by the stand-alone spICP-MS would not accurately reflect the true sizes of the Fe-containing NPs with more complex compositions. For example, the major species of Fe-containing NPs present in tap water due to corrosion of iron components in the distribution system are likely to be iron oxides and iron hydroxides such as Fe_3_O_4_, Fe_2_O_3_, and FeOOH,^43,44^ which possess a larger size than elemental iron particles due to the Fe–O coordination. The larger mode size (*D*_e_ = 110 nm) determined by the ATM-DMA-spICP-MS hyphenated system reflected the fact that the Fe-containing NPs were not pure elemental iron particles and better represented the size of the Fe-containing NPs in the drinking water sample. The particle number concentration determined by the stand-alone spICP-MS and the hyphenated system were 1.44 × 10^7^ and 4.99 × 10^7^ #/mL, respectively. The lower particle number concentration obtained by the stand-alone spICP-MS was likely due to errors introduced in the dilution procedures. The particle number concentration obtained using the hyphenated system did not exceed its upper quantification limit (4.0 × 10^8^ #/mL). Due to the higher upper limit of particle number concentration, no dilution was needed for this tap water sample, which could avoid potential errors introduced in the dilution procedure. Still, dilution is needed for samples with a relatively high particle number concentration in ATM-DMA-spICP-MS analysis.

For Pb-containing NPs, the mode sizes determined by the stand-alone spICP-MS and the hyphenated system were 21 nm (*D*_m_) and 100 nm (*D*_e_), respectively (Table 2). Again, the smaller sizes determined by the stand-alone spICP-MS may not accurately reflect the true sizes of the Pb-containing NPs with more complex compositions. Pb corrosion products, including carbonates, such as cerussite (PbCO_3_) and hydrocerussite (Pb_3_(CO_3_)_2_(OH)_2_), and oxides, such as litharge (PbO) and scrutinyite/plattnerite (PbO_2_), could be present in the distribution system.^38−40,45^ Detachments of these corrosion particles could contribute to the Pb-containing NPs in drinking water.^12,41,42,46^ Therefore, the mode size (*D*_e_ = 100 nm) determined by the ATM-DMA-spICP-MS hyphenated system should better represent the size of the Pb-containing NPs in the drinking water sample. The particle number concentration determined by the stand-alone spICP-MS and the hyphenated system were 3.99 × 10^6^ and 1.40 × 10^7^ #/mL, respectively. The reason for the relatively low particle number concentration obtained from the stand-alone spICP-MS could be the same as that for Fe-containing NPs. Overall, the ATM-DMA-spICP-MS hyphenated system provided a more reliable quantification of the particle size and particle number concentration of Fe-containing and Pb-containing NPs in the tap water sample.

It should be noted that Fe-containing and Pb-containing NPs investigated in this study are predominately present as oxides, hydroxides, and carbonates, in which Fe or Pb is the sole metallic element in these NPs. If the NPs contain more than one metallic element, spICP-TOF-MS should be employed to provide the information of elemental compositions of each individual NP.^47−50^

## 4 Environmental Implications

The ATM-DMA-spICP-MS hyphenated system was employed for characterizing Fe-containing and Pb-containing NPs in tap water. As demonstrated using the reference AuNPs and Ag-shelled AuNPs for validation purposes, the hyphenated system with proper sample heating could accurately determine the size of pure and nonpure metallic NPs, which could not be achieved using the stand-alone spICP-MS. For tap water analysis, the mode sizes and particle number concentrations of Fe-containing NPs and Pb-containing NPs determined by the hyphenated ATM-DMA-spICP-MS system could better reflect the true sizes and particle number concentrations of these NPs after the sample was centrifugated to remove soluble ions. The proposed method can be used in the assessment of human exposure to different metallic NPs due to drinking water consumption. The application of this method to a more complex water matrix such as natural water and wastewater with high organic contents warrants further studies.

## Supporting Information Available

Transport efficiency determination for the stand-alone spICP-MS; transport efficiency determination for the ATM-DMA-spICP-MS hyphenated system; determination of upper limit particle number concentration for the stand-alone spICP-MS; determination of upper limit particle number concentration for the ATM-DMA-spICP-MS hyphenated system; mode sizes and particle number concentrations of Fe-containing NPs and Pb-containing NPs analyzed by stand-alone spICP-MS with different dilution factors; TEM images of AuNPs and Ag-shelled AuNPsize of AuNPs; and influence of centrifugation on the frequency detected in the ATM-DMA-spICP-MS hyphenated system (PDF)
