# Supplementary material for: Characterization of Fe-Containing and Pb-Containing Nanoparticles Resulting from Corrosion of Plumbing Materials in Tap Water Using a Hyphenated ATM-DMA-spICP-MS System
Source: Environ Sci Technol. 2024 Jan 19;58(4):2038–47. doi: 10.1021/acs.est.3c07592 (PMC10832032; doi:10.1021/acs.est.3c07592)
Supplement: Supplementary file 1 — es3c07592_si_001.pdf [file es3c07592_si_001.pdf]

Supporting Information

Characterization of Fe-containing and Pb-containing nanoparticles resulting from corrosion of plumbing materials in tap water using a hyphenated ATM-DMA-spICP-MS system

Jing-Wen Wang[1], Chia-Hung Yu[1], Wen-Che Hou[2], Ta-Chih Hsiao[1]*, Yi-Pin Lin[1,3]*

[1]Graduate Institute of Environmental Engineering, National Taiwan University, No. 1, Sec. 4, Roosevelt Road, Taipei 10617, Taiwan

[2]Department of Environmental Engineering, National Cheng Kung University, No. 1 University Road, Tainan City 70101, Taiwan

[3]NTU Research Center for Future Earth, National Taiwan University, No. 1, Sec. 4, Roosevelt Road, Taipei 10617, Taiwan

*Corresponding Author, Phone: + 886-2-3366-4401, E-mail: tchsiao@ntu.edu.tw

Phone: +886-2-3366-4380, E-mail: yipinlin@ntu.edu.tw

8 pages

5 Tables

2 Figures

Table S1. Transport efficiency determination (η) for the stand-alone spICP-MS (n=23). 50 nm AuNPs reference solution was used and the scan time (t) of the spICP-MS is 0.5 min. η= $\frac{(N/Qt)}{C_n}$.

Introduced particle number concentration Cn (#/mL)	Liquid sample flow rate Q (mL/min)	Counting N(#)	Transport efficiency η (%)
4×10^4	0.355	426	6.00
4×10^4	0.332	400	6.02
4×10^4	0.330	397	6.02
4×10^4	0.320	387	6.05
4×10^4	0.323	372	5.76
4×10^4	0.299	559	9.34
4×10^4	0.330	397	6.02
4×10^4	0.330	512	7.75
4×10^4	0.332	506	7.62
4×10^4	0.320	380	5.93
4×10^4	0.390	498	6.39
4×10^4	0.370	558	7.54
4×10^4	0.343	346	5.05
4×10^4	0.362	484	6.68
4×10^4	0.370	603	8.15
4×10^4	0.364	374	5.14
4×10^4	0.310	288	4.64
4×10^4	0.300	311	5.19
4×10^4	0.380	426	5.61
4×10^4	0.320	371	5.79
4×10^4	0.332	275	4.14
4×10^4	0.303	354	5.84
4×10^4	0.325	495	7.62

Table S2. Transport efficiency determination (η) for the ATM-DMA-spICP-MS hyphenated system (n=20). 50 nm AuNPs reference solution was used and the scan time (t) of the spICP-MS is 0.5 min. $\eta = \frac{(N/Qt)\times D}{C_n}$. D is a parameter considering the sample dilution in ATM, DMA and spICP-MS. D= 38.53 L gas/mL liquid.

Introduced particle number concentration C_n (#/mL)	Gas ample flow rate Q (L/min)	Counting N(#)	Transport efficiency η (%)
1×10^7	0.3	2418	1.55
1×10^7	0.3	2717	1.74
1×10^7	0.3	2892	1.86
2×10^7	0.3	4663	1.50
2×10^7	0.3	5315	1.71
2×10^7	0.3	5183	1.66
4×10^7	0.3	7624	1.22
4×10^7	0.3	7428	1.19
4×10^7	0.3	7326	1.18
4×10^7	0.3	7459	1.20
4×10^7	0.3	8915	1.43
4×10^7	0.3	8960	1.44
4×10^7	0.3	9075	1.46
4×10^7	0.3	6364	1.02
4×10^7	0.3	6705	1.08
4×10^7	0.3	8165	1.31
4×10^7	0.3	8075	1.30
1×10^8	0.3	15513	1.00
1×10^8	0.3	16394	1.05
1×10^8	0.3	16747	1.08

Table S3. The determination of upper limit particle number concentration for the stand-alone spICP-MS.

Introduced particle number concentration (#/mL)	Mode size D_m (nm)	Frequency (#)	Transport efficiency (%)	Obtained particle number concentration (#/mL)	Recovery (%)
1×10^5	52	808	5.14	9.82×10^4	98.2
1×10^5	53	731	5.05	9.05×10^4	90.5
1×10^5	53	903	6.05	9.33×10^4	93.3
2×10^5	58	1689	7.12	1.49×10^5	75.0
2×10^5	54	1806	6.05	1.87×10^5	83.5
2×10^5	58	1706	6.05	1.76×10^5	88.0
4×10^5	64	3172	7.12	2.78×10^5	69.5
4×10^5	63	3227	7.12	2.83×10^5	70.8
4×10^5	66	3150	7.12	2.77×10^5	69.3

Table S4. The determination of upper limit particle number concentration for the ATM-DMA-spICP-MS hyphenated system.

Introduced particle number concentration (#/mL)	Mode size D_e (nm)	Frequency (#)	Transport efficiency (%)	Obtained particle number concentration (#/mL)	Recovery (%)
4 x 10^6	53	1352	1.76	4.93 x 10^6	123.3
1 x 10^7	52	2418	1.55	1.00 x 10^7	100.1
2 x 10^7	53	4663	1.50	2.00 x 10^7	100.2
4 x 10^7	54	7624	1.22	4.01 x 10^7	100.3
1 x 10^8	54	16748	1.08	9.96 x 10^7	99.6
2 x 10^8	54	33836	1.04	2.09 x 10^8	104.5
4 x 10^8	66	62061	0.83	5.20 x 10^8	130.0

Table S5. Mode sizes and particle number concentrations of Fe-containing NPs and Pb-containing NPs analyzed by stand-alone spICP-MS with different dilution factors.

	Dilution Factor	Mode Size (nm)	Particle Number Concentration (#/mL)
Fe-containing NPs	1	90	$4.13x10^4$
	50	40	$5.82\ x10^6$
	100	36	$7.09\ x10^6$
	200	35	$7.26\ x10^6$
Pb-containing NPs	1	47	$5.86\ x10^3$
	50	31	$5.41\ x10^5$
	100	26	$2.19\ x10^6$
	200	26	$2.34\ x10^6$

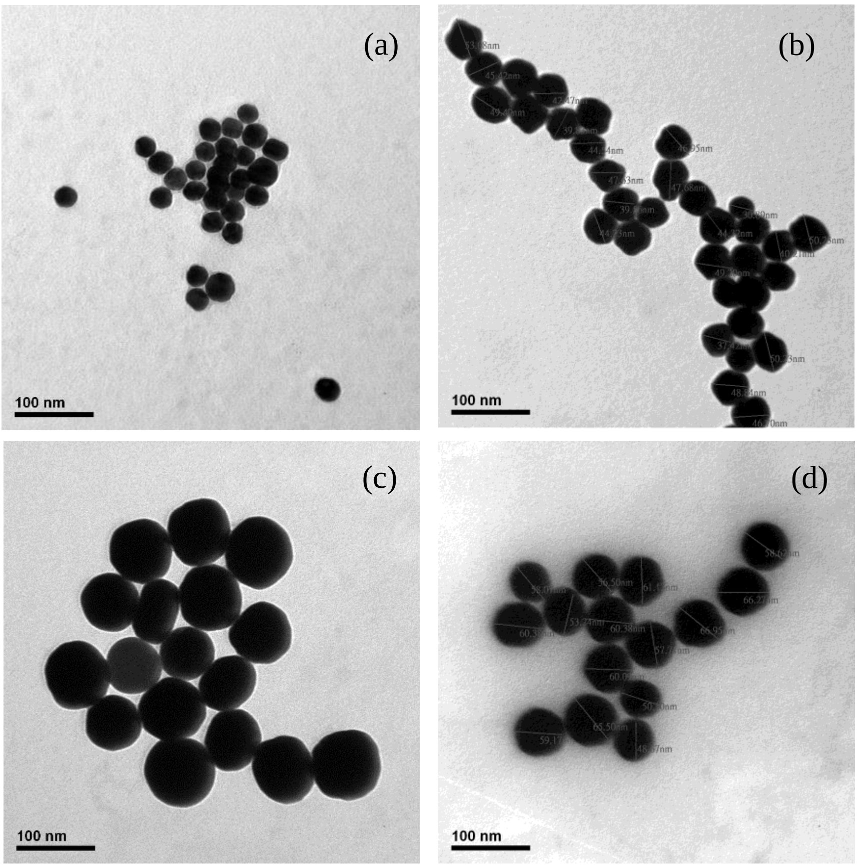

Figure S1. TEM images (a) 30 nm AuNPs, diameter = 29.5 ± 3.1 nm (N= 23) (b) 50 nm AuNPs, diameter = 45.1 ± 4.5 nm (N= 33) (c) 80 nm AuNPs, diameter = 77.6 ± 7.0 nm (N= 17) (d) 60 nm Ag-shelled AuNP, diameter = 58.9 ± 5.3 (N=15)

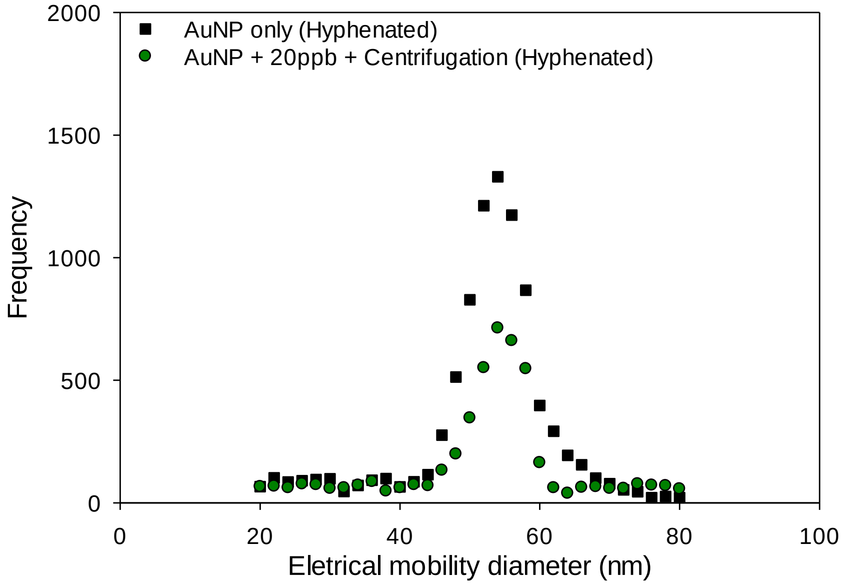

Figure S2. Influence of centrifugatiuon on the frequency detected in the ATM-DMA-spICP-MS hyphenated system
